# DP71 and SERCA2 alteration in human neurons of a Duchenne muscular dystrophy patient

**DOI:** 10.1186/s13287-018-1125-5

**Published:** 2019-01-15

**Authors:** Simona Ruggieri, Luigi Viggiano, Tiziana Annese, Carmela Rubolino, Andrea Gerbino, Roberta De Zio, Patrizia Corsi, Roberto Tamma, Domenico Ribatti, Mariella Errede, Francesca Operto, Lucia Margari, Nicoletta Resta, Silvia Di Tommaso, Jessica Rosati, Maria Trojano, Beatrice Nico

**Affiliations:** 1 Department of Basic Medical Sciences, Neurosciences and Sensory Organs, University of Bari Medical School, Bari, Italy; 2 Department of Biology, University of Bari, Bari, Italy; 3 Department of Bioscience, Biotechnology and Biopharmaceutics, University of Bari, Bari, Italy; 4 Division of Medical Genetics, Department of Biomedical Sciences and Human Oncology (DIMO), University of Bari Medical School, Bari, Italy; 5 Cellular Reprogramming Unit, IRCCS Casa Sollievo della Sofferenza, San Giovanni Rotondo, Italy; 6 Department of Biomedical Sciences and Human Oncology, Unit of Internal Medicine “Guido Baccelli”, University of Bari Medical School, Bari, Italy

**Keywords:** Duchenne muscular dystrophy, Dp71 dystrophin, Pluripotent stem cell, SERCA2, hiPSCs, Neurons

## Abstract

Cognitive deficit has been identified in one third of patients affected by Duchenne Muscular Dystrophy, primarily attributed to loss of the short Dp71 dystrophin, the major brain dystrophin isoform. In this study, we investigated for the first time the Dp71 and Dp71-associated proteins cellular localization and expression in human neurons obtained by differentiation from induced pluripotent stem cell line of a patient affected by cognitive impairment. We found structural and molecular alterations in both pluripotent stem cell and derived neurons, reduced Dp71 expression, and a Ca^2+^ cytoplasmic overload in neurons coupled with increased expression of the SERCA2 pump in the dystrophic neurons. These results suggest that the reduction of Dp71 protein in the Duchenne muscular dystrophy neurons leads to alterations in SERCA2 and to elevated cytosolic Ca^2+^ concentration with consequent potential disruption of the dystrophin proteins and Dp71-associated proteins.

**Electronic supplementary material:**

The online version of this article (10.1186/s13287-018-1125-5) contains supplementary material, which is available to authorized users.

## Background

Mutations in the *dmd* gene are responsible for the Duchenne muscular dystrophy (DMD) disease [1], in which muscular degeneration is also associated with cognitive defects likely due to loss of the smallest product of the *dmd* gene, the Dp71 dystrophin isoform [2]. Dp71 is the most abundant dystrophin gene product in the adult brain [3, 4], and DMD patients with Dp71 partial ablation display severe mental retard with a reduction of the mean intelligence quotient (IQ) by about 1 standard deviation [5]. In the CNS, Dp71 is detected in neurons [6], but little is known about its function for no availability of DMD patient’s brain tissues. Differentiation of hiPSCs into neural cells provides a recent technology to generate living neurons genetically identical to the patients’ ones. In this study, we have used the hiPSCs obtained from a DMD patient affected by serious cognitive impairment, to generate the correspondent neuronal lineage with the aim to establish a cell model to disclose the role of the Dp71 in the neuronal alterations associated with DMD. We have performed ultrastructural characterization and expression analysis for Dp71 and DAPs including AQP4 and βDG in both DMD hiPSCs and neurons. Moreover, to better understand the mechanism leading to alterations of neuronal cells in DMD brain, we have investigated the relationship between Dp71 and SERCA2 in DMD neurons. We evaluated the permeability of the endoplasmic reticulum (ER) blocking the SERCA2 pump by the specific inhibitor CPA. The results showed structural and molecular alterations in hiPSCs and neurons cells, a reduction of Dp71, and DAPs transcription and transduction. Interestingly, intracellular Ca^2+^ homeostasis was impaired in dystrophic neurons suggesting that, as in the dystrophic muscle [7, 8], an abnormal increase of intracellular Ca^2+^ could be involved as early pathogenic event of mental retard in DMD.

## Materials and methods

Detailed methods are included in Additional file 1.

### Case report

A 22-year-old patient shows a severe motor impairment; his total intelligence quotient (IQ), assessed with Wechsler Intelligence Scale for Children–Fourth Edition (WISC-IV), is 68, confirming the mild intellectual disability previously diagnosed (Additional file 1).

### Generation of DMD hiPSCs

We have collected samples of PBMCs from a DMD patient and healthy donor. The PBMCs were reprogrammed to obtain the hiPSCs line as described in Additional file 1.

### Neuronal differentiation

hiPSCs were maintained in supplemented mTeSR medium (Stem Cell Technologies) under standard conditions. hiPSCs were differentiated to glutamatergic sensory neurons using a previously described protocol [9, 10] (Additional file 1).

### Electron microscopy

The hiPSCs and neurons cells were fixed and embedded in Epon 812. Semithin and ultrathin sections were performed as described in Additional file 1.

### Dual immunofluorescence confocal laser scanning microscopy and morphometric analysis

hiPSCs and neuronal cells were fixed and then exposed to primary and corresponding secondary antibodies (Additional file 1: Table S1). The cells were examined under a Leica TCS SP2 (Leica, Wetzlar, Germany) confocal laser scanning microscope (Additional file 1).

### Real-time PCR

The expression of mRNA for Dp71, AQP4, Dys, DG, and SERCA2 was evaluated by real-time PCR (Chromo4 Real-Time PCR Detection System - Bio-Rad Laboratories), and samples were normalized to *cyclophylin-A* as housekeeping gene (Additional file 1).

### Western blotting

The protein content of Dp71, AQP4, Dys, DG, and SERCA2 was evaluated by immunoblotting analysis (Additional file 1).

### Electrophysiological measurements

Both control and patient’s iPSC-derived glutamatergic neurons, plated on 35-mm culture dishes, were recorded at room temperature. Whole cell experiments were performed with a Multiclamp 700B amplifier (Axon CNS-Molecular Devices, Sunnyvale, CA, USA) connected to an Axon Digidata 1500 (Axon Instrument-Molecular Devices, Sunnyvale, CA, USA). Currents were sampled at 10 kHz and low pass filtered at 5 kHz (Additional file 1).

### Intracellular Ca^2+^ measurements

Cells were seeded on matrigel-coated glass coverslips (Ø 35 mm). Cells were loaded with 2–4 μM Fluo-4 (Thermo Fisher Scientific, Waltham, MA, USA) 25 min at 37 °C in DMEM. Coverslips with dye-loaded cells were mounted in a perfusion chamber (FCS2 Closed Chamber System, BIOPTECHS, Butler, USA), and measurements were performed using an inverted microscope (Nikon Eclipse TE2000-S microscope) equipped for single-cell fluorescence measurements and imaging analysis (Additional file 1).

### Statistics

Data are reported as means ± SEM. Student’s *t* test was used for two-group comparisons, and Bonferroni post-test was used to compare all treatment groups following one-way ANOVA. The Graph Pad Prim 5.0 statistical package (GraphPad Software, San Diego, CA, USA) was used for the analysis, and *P* < 0.05 values were considered statistically significant.

## Results

### Ultrastructural and morphological alterations of DMD-hiPSCs

**Fig. 1:**
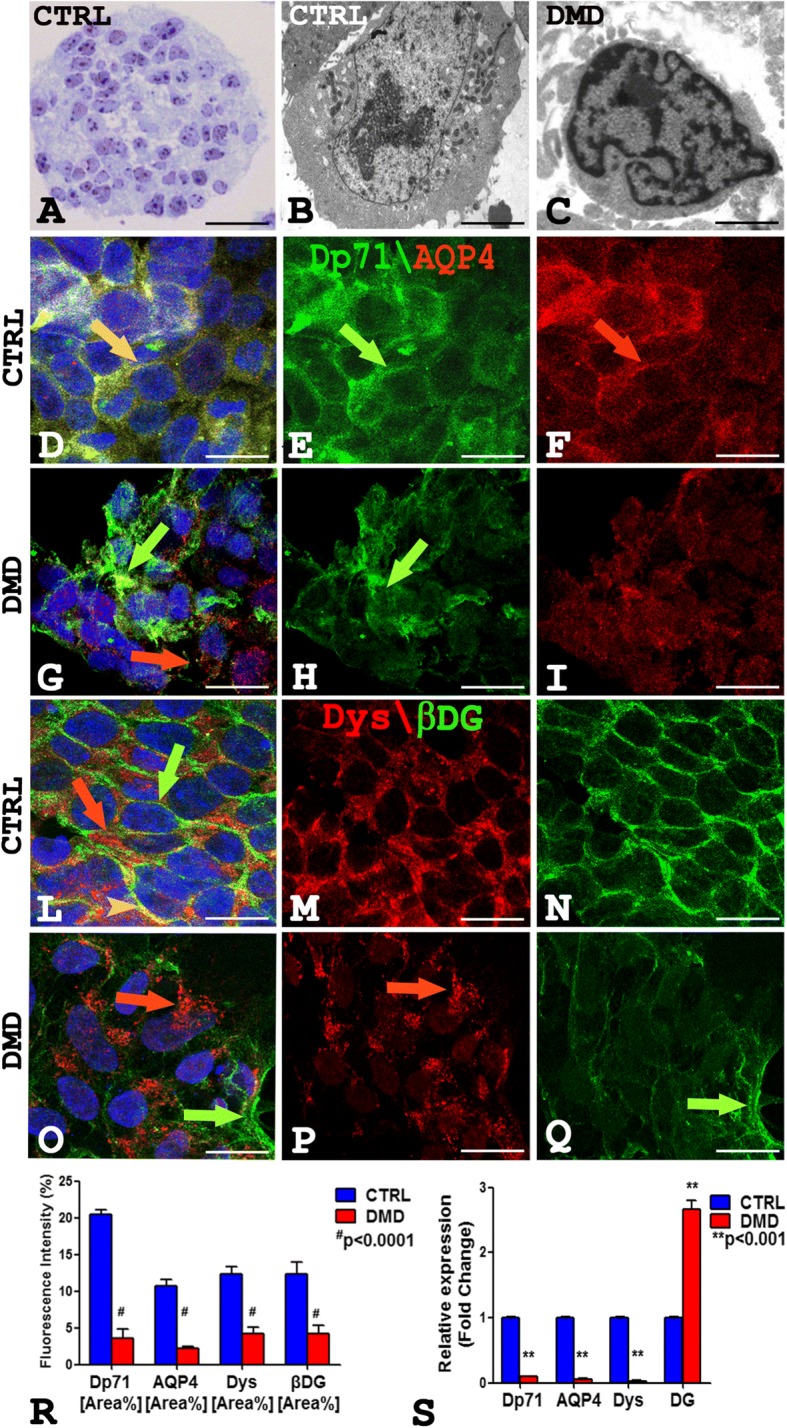
**a**–**c** Semithin CTRL section stained with toluidine blue (**a**) showing a rounded neurosphere with healthy cells, and ultrathin section (**b**, **c**) of hiPSCs showing a larger CTRL hiPSCs with abundant mitochondria and organelles (**b**) compared to DMD hiPSCs with heterocromatic and irregular nucleus (**c**). **d**–**i** Dp71 green and AQP4 red confocal immunofluorescence show an intensity fluorescence reduction in DMD hiPSCs (**g**–**i**) compared to the control ones (**d**–**f**). Dp71 green signal is membrane disarranged and scattered in the cytoplasm in DMD hiPSCs (**h** arrow). An orange fluorescence colocalization of Dp71 and AQP4 is present in control cells (**d** yellow arrow) while separate fluorescent signals are present in DMD cells (**g** green and red arrows). **l**–**q** Dys red and βDG green confocal immunofluorescence display a Dys pointed cytoplasm signals (**o**, **p** red arrow) and disarranged βDG green fluorescent in DMD hiPSCs (**o**, **q** green arrow) compared to strong and polarized signals in the controls (**l**–**n** red arrow, green arrow). A merge orange signal for Dys and βDG is present in short membrane tracts in CTRL hiPSCs (**l** orange arrowhead) while separate signals are present in DMD hiPSCs (**o** green and red arrows). Morphometric analysis (**r**) shows a significant Dp71, AQP4, Dys, and βDG fluorescence intensity reduction in DMD compared to CTRL cells. The mRNA expression analysis (**s**) shows reduction of Dp71, AQP4, Dys mRNA, and increased of DG mRNA in the DMD cells compared with control. Scale bar: **a** 30 μm; **b**, **c** 2.5 μm; **d**–**q** 7.5 μm. Data are represented as mean ± SEM

On the semithin section of both control and patient, the neurospheres appeared rounded shape (Fig. 1a), while on the ultrathin sections, the hiPSCs showed ultrastructural differences. Control hiPSCs (Fig. 1b) appeared larger (50% more) with abundant mitochondria and organelles compared to the DMD-hiPSCs that showed heterochromatic and irregular nucleus (Fig. 1c). Morphometric analysis (Additional file 1: Figure S2) displayed a significant reduction of the DMD-hiPSCs surface area (29.35 ± 4.851SEM) and perimeter (15.69 ± 0.7255 SEM) compared to the control ones (107.9 ± 9.619 SEM; 31.85 ± 2.776 SEM).

The dual confocal immunofluorescence reaction on DMD-hiPSCs for Dp71 and AQP4, Dp427 dystrophin (Dys) and βDG (Fig. 1d–q) showed the reduction of the proteins (Fig. 1g–i, o–q) while a strong fluorescence signal was present in the control (Fig. 1d–f, l–n). The distribution pattern of the Dp71 and AQP4 proteins changed in the patient’s hiPSCs (Fig. 1g–i) compared to the control (Fig. 1d–f).

The Dp71 protein was membrane disarranged and scattered in the cytoplasm (Fig. 1h), and separate Dp71 and AQP4 fluorescence signal was detected in DMD (Fig. 1g). Otherwise, Dp71 and AQP4 strongly colocalized on the membrane and cytoplasm of control (Fig. 1d). Furthermore, DMD-hiPSCs displayed a strong molecular rearrangement of Dys and βDG proteins with a dystrophin pointed fluorescence signals in cytoplasm (Fig. 1o, p) and reduced βDG green fluorescence, while a strong and polarized fluorescence in control ones was observed (Fig. 1l–n). Moreover, Dys and βDG colocalized in a short membrane tract only in the control hiPSCs (Fig. 1l). Morphometric analysis shows a meaningful reduction of Dp71, AQP4, Dys, and βDG fluorescence intensity in DMD-hiPSCs compared to controls (Fig. 1r). Real-time PCR experiments revealed a significant reduction in mRNA amount of Dp71, AQP4, Dys, and an increment of the messenger for DG in DMD-hiPSCs compared to control (Additional file 1: Figure S1). In parallel, immunoblotting experiments show a significant reduction of the Dp71, AQP4, Dys, and βDG protein content (Additional file 1: Figure S4).

### Ultrastructural and morphological alterations of DMD neurons

**Fig. 2:**
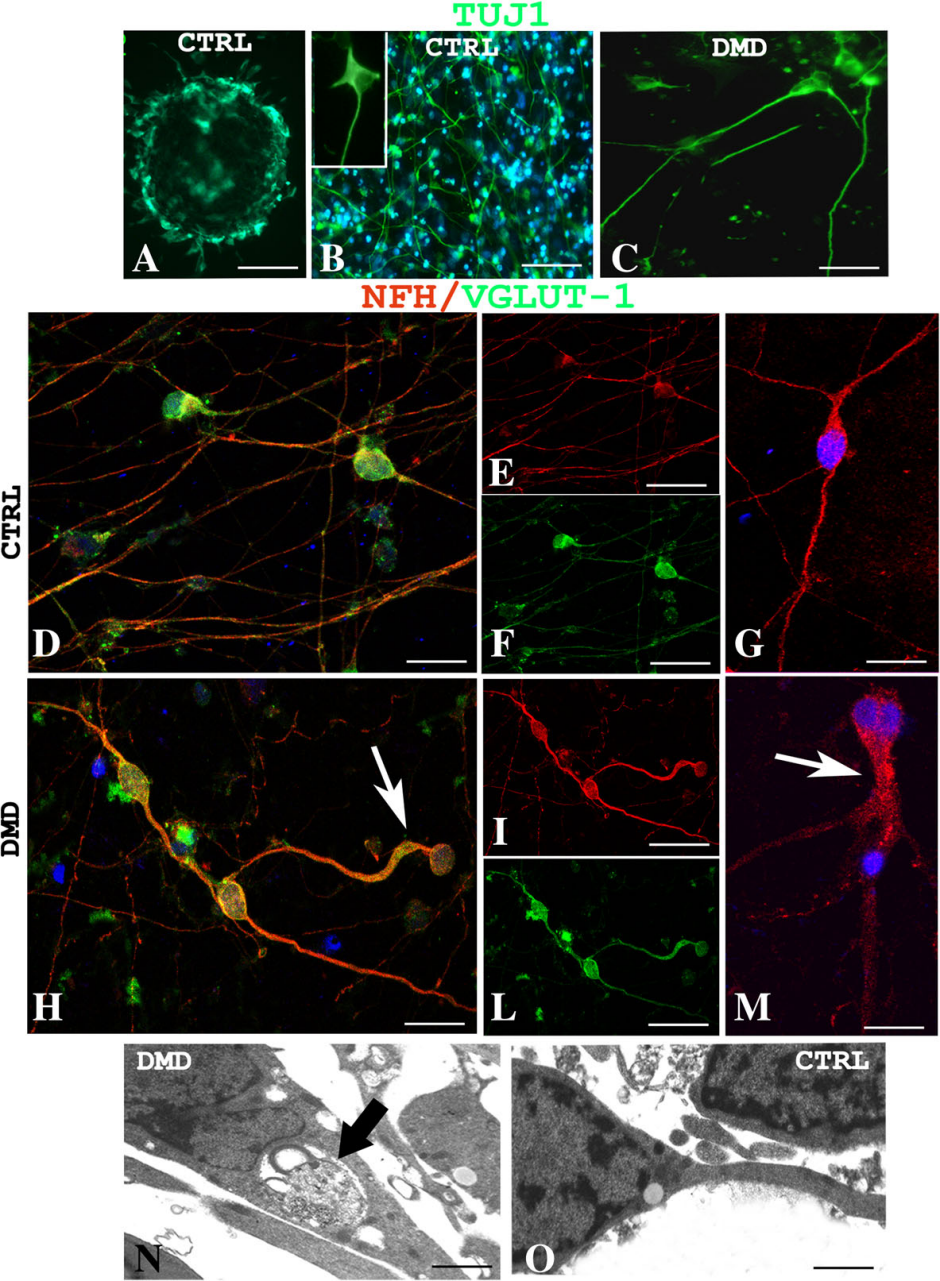
Confocal immunofluorescence reaction of TuJ-1 in CTRL (**a**, **b**) and DMD (**c**) neurons reveals prevalently multipolar control neurons (**b** inset) and unipolar and bipolar DMD neurons (**c**). VGLUT-1(green) and NFH (red) dual immunofluorescence shows DMD abnormal neurons with spread out processes (**h**–**m**, arrows) compared with the control ones (**d**–**g**). Neurons of both CTRL and DMD (**d**, **h**) display an orange fluorescence signal of VGLUT and NFH colocalization. Ultrastructural analysis shows DMD neurons with dilated processes containing autophagic vacuoles (**n** arrow), differently to healthy control neurons (**o**). Scale bar: **a** 40 μm; **b** 60 μm; **c** 20 μm; **d**, **g**, **h**, **m**, 10 μm; **e**, **f**, **i**, **l** 20 μm; **n**, **o** 0.5 μm

**Fig. 3:**
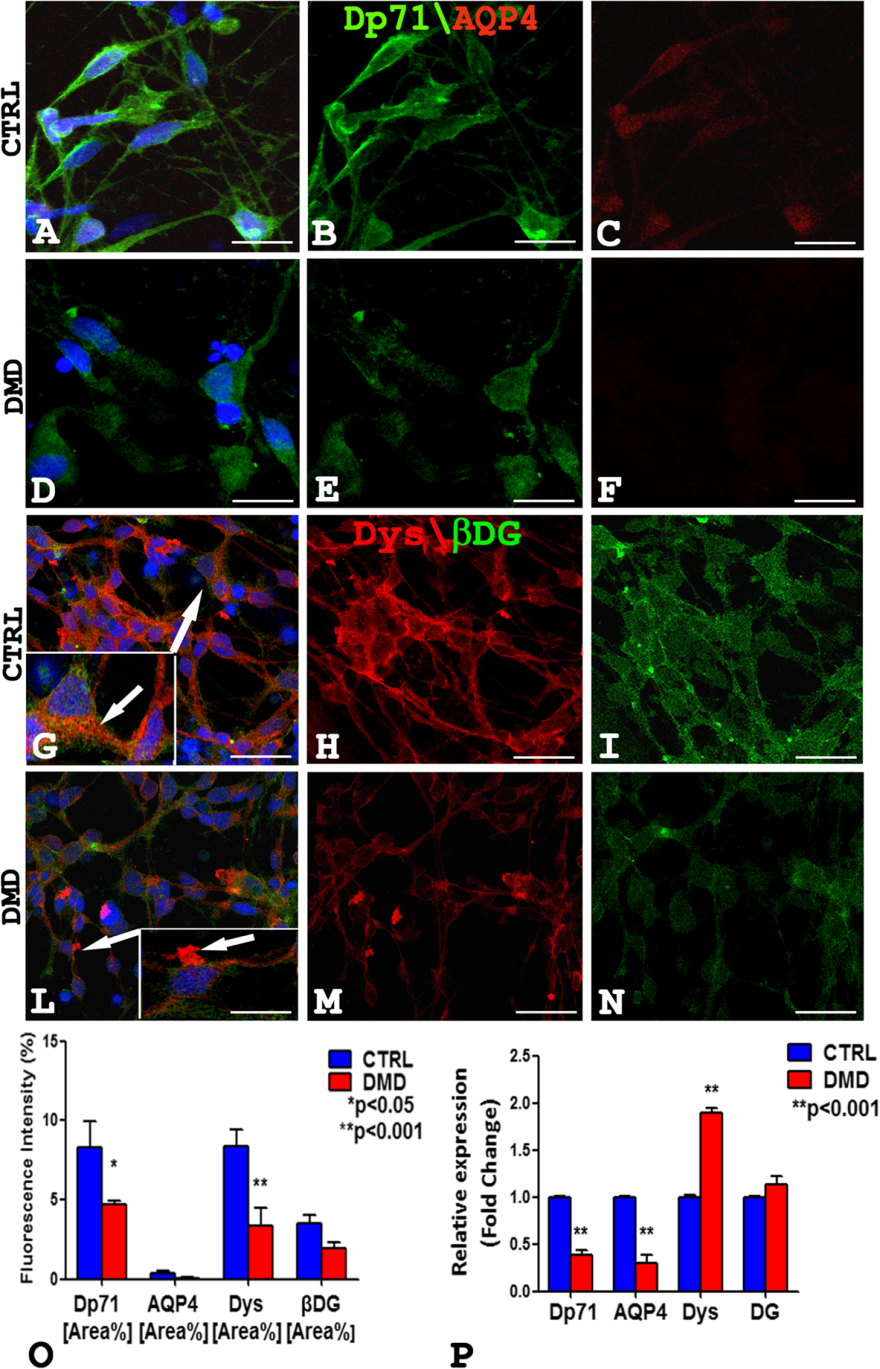
**a**–**f** The Dp71 (green) and AQP4 (red) confocal analysis shows the reduction of Dp71 fluorescence in DMD-neurons (**d**, **e**) compared with a strong Dp71 signal in the control ones (**a**, **b**) while the AQP4 fluorescence is missing in DMD neurons (**d**, **f**) and weakly expressed in the control (**a**, **c**). **g**–**n** Dys (red) and βDG (green) dual reaction shows a lower βDG signal in DMD neurons (**l**, **n**) compared to the control ones (**g**, **i**) and a Dys clustering in DMD cytoplasm (**l** and inset arrow, **m**), and a diffused signal in the control cytoplasm (**g** and inset arrow, **h**). **o** Morphometric analysis shows a significant reduction of Dp71 and Dys fluorescence intensity in DMD neurons compared to control. **p** The mRNA expression analysis shows reduction of Dp71, AQP4 mRNA, and an increase of DG and Dys mRNA in DMD neurons compared to control. Scale bar: **a**–**f** 12 μm; **g**–**n** 20 μm. Data are represented as mean ± SEM

Control and patient’s hiPSCs were differentiated in mature glutamatergic sensory neurons [9]. Of note, both control and DMD-differentiated neurons were able to generate action potentials (Additional file 1: Figure S3). To identify the neurons, a TuJ-1 immunofluorescence reaction was performed (Fig. 2a). Noteworthy, control neurons were multipolar (Fig. 2b), whereas DMD neurons were prevalently unipolar and bipolar (Fig. 2c). To further characterize the neurons, a dual immunofluorescence reaction with antibodies anti-vesicular glutamate transporter-1 (VGLUT-1) and anti-neurofilament (NFH) was performed (Fig. 2d–m). The neurons, in both DMD and control patient, displayed an orange fluoresce signal of VGLUT-1 and NFH colocalization (Fig. 2d–f, h–l). Morphometric analysis showed no significant differences (*P* > 0.05) of VGLUT-1 and NFH fluorescence intensity between DMD (mean 4.04 ± SEM 0.17; mean 4.96 ± SEM 0.57) and control (mean 3.35 ± SEM 0.30; mean 6.40 ± SEM 0.47) neurons (data not shown). DMD neurons appeared structurally abnormal showing neuronal processes strongly spread out (Fig. 2h, m, arrow) compared with control ones (Fig. 2d–g). Ultrastructural analysis confirmed the alterations of DMD-neurons showing dilated processes containing autophagic vacuoles (Fig. 2n) compare to control neurons (Fig. 2o). Moreover, the confocal analysis for Dp71 and AQP4 showed the reduction of Dp71 protein in DMD neurons (Fig. 3d, e) compared to strong Dp71 expression in the control ones (Fig. 3a, b) while the AQP4 protein was lacking in DMD neurons (Fig. 3d, f) and weakly expressed in control one (Fig. 3a, c). Furthermore, Dys and βDG dual reaction showed a lower expression of the βDG on patient’s neurons (Fig. 3l, n) compared to the control ones (Fig. 3g, i) and a strong reduction in Dys expression with a clustered signal in DMD cytoplasm (Fig. 3l, m), while a diffuse signal in control cytoplasm (Fig. 3g, h) was detected. No Dys and βDG colocalization was observed in both control and DMD neurons (Fig. 3g, l). The confocal observations were confirmed by morphometric analysis (Fig. 3o). After qPCR, a significant reduction in the expression of mRNA coding for Dp71 and AQP4 and an increase of the messenger for DG and Dys were observed in DMD neurons when compared to control (Fig. 3p).

In parallel, immunoblotting experiments show a significant reduction of the Dp71, AQP4, Dys, and βDG protein content (Additional file 1: Figure S4).

### Alteration in SERCA2 and Dp71 expression and abnormal Ca^2+^ homeostasis in DMD-hiPSC-derived neurons

**Fig. 4:**
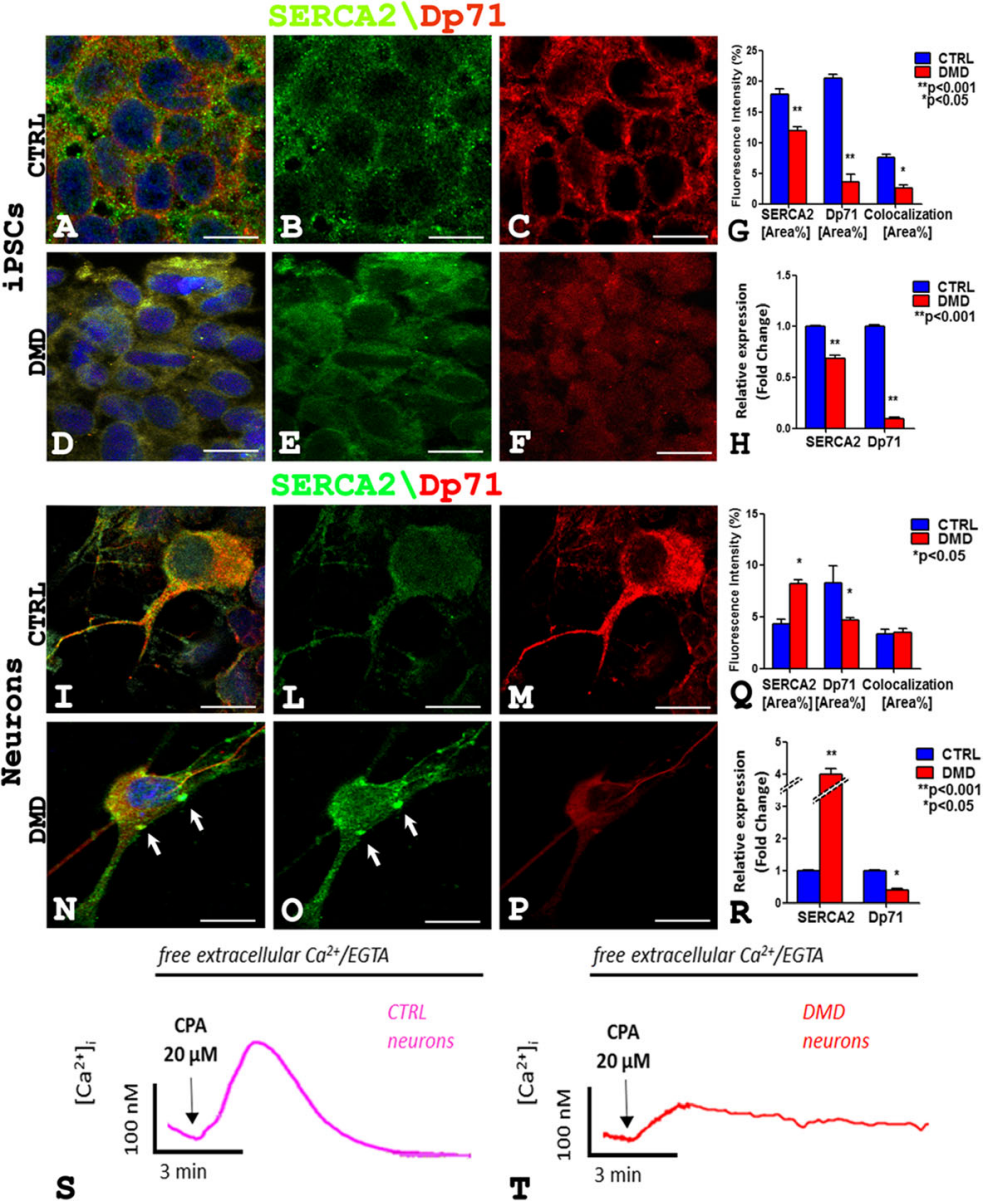
SERCA2 (green) and Dp71 (red) fluorescence signals decrease and diffuse in DMD hiPSCs cytoplasms (**d**–**f**) compared to intense membrane expression in the control (**a**–**c**) while SERCA2 signal increases (**o**) and DP71 decreases (**p**) in DMD neurons (**n**–**p**) compared to control ones (**i**–**m**). An orange fluorescence signal of colocalization for SERCA2 and Dp71 is present in hiPSCs (**a**, **d**) and neurons (**i**, **n**) of both DMD and CTRL. Note a concentrate point SERCA2 expression (**n**, **o** arrow) in DMD neuron. Morphometric analysis (**g**, **q**) shows a significative reduction of SERCA2 and Dp71 fluorescence intensity in hiPSCs in DMD (**g**) and significative SERCA2 increase and DP71 decrease in DMD neurons (**q**). The mRNA expression analysis (**h**, **r**) shows reduction of SERCA2 and Dp71 mRNA in DMD hiPSCs (**h**) and mRNA SERCA2 increase and DP71decrease in DMD neurons (**r**) compared to the control. Experiments of ER Ca^2+^-depletion kinetics show significative reduction of CPA-induced ER Ca^2+^ release in DMD neurons (**t**) compared to CTRL neurons (**s**). Scale bar: **a**–**f**, **i**–**p** 7.5 μm. Data are represented as mean ± SEM

To investigate the mechanisms involved in the mental retard in DMD, we investigated SERCA2 expression in hiPSCs and in neurons of the patient and control. SERCA2 and Dp71 dual confocal reaction highlights that in DMD-hiPSCs (Fig. 4a–f) there is a decrease of SERCA2 and Dp71 amount (Fig. 4d–f) compared to the control (Fig. 4a–c). Instead, the neurons (Fig. 4i–p) show an increase of SERCA2 (Fig. 4n, o) and a decrease of Dp71 (Fig. 4n, p) content in DMD (Fig. 4n–p) compared to the control (Fig. 4i–m). Moreover, SERCA2 and Dp71 colocalization was observed both in hiPSCs (Fig. 4a, d) and in neurons (Fig. 4i, n) in the patient (Fig. 4d, n) and control (Fig. 4a, i). These observations were confirmed by morphometric analysis, qPCR (Fig. 4g, q, h, and r), and western blotting analysis (Additional file 1: Figure S4).

This increased expression of the SERCA pump in DMD neurons could likely shift the dynamic equilibrium between ER Ca^2+^ accumulation and Ca^2+^ passive leak toward a higher level of intraluminal Ca^2+^. Therefore, we evaluated SERCA2 activity as the passive Ca^2+^ permeability of the ER when the Ca^2+^-ATPase is blocked by CPA. This is a well-known experimental procedure that will increase cytosolic Ca^2+^ level as index of ER Ca^2+^ levels [11, 12]. In Fig. 4s, t, two representative experiments of ER Ca^2+^-depletion kinetics in Fluo-4 loaded control and DMD-neurons are shown. The cells were treated with the SERCA blocker (20 μM CPA) while perfused with Ca^2+^-free medium. Of note, CPA-induced ER Ca^2+^ release was significantly reduced in DMD neurons (Fig. 4t) when compared with control cells (Additional file 1: Figure S4). This evidence does not correlate with the increased expression level of the SERCA pump in DMD neurons and could indicate the fact that in the ER of DMD neurons, the intraluminal Ca^2+^ levels are lower than in the ER of control neurons as previously reported for muscle tissue [13].

## Discussion

The investigation of DMD neuropathogenesis in human is hampered by the absence of a satisfactory human model. In the present study, we have used the hiPSC technology to generate three glutamatergic sensory neuronal lineages of DMD patient, and we have obtained a cell model able to identify morphostructural and functional alterations in patient’s hiPSCs and neurons cells. The results demonstrated that Dp71 and DAPs alterations were already present in dystrophic stem cells as in neurons and even if some genes were overexpressed, such as βDG in hiPSCs and βDG and Dys in neurons, Dp71 and DAPs proteins were reduced and disarranged in DMD hiPSCs and neurons. We hypothesize that the reduction in Dp71 and DAPs proteins content could be related to activation of ubiquitin-proteasome complex as demonstrated in our previous work on *mdx* mice [14]. Moreover, the DMD hiPSCs and neurons showed ultrastructural modification similar to that observed in *mdx* mesenchymal stem cells [15]. DMD is characterized by intracellular Ca^2+^ ([Ca^2+^]i) dyshomeostasis in skeletal and cardiac muscles [16–19]. The association between the lack of dystrophin and intracellular Ca^2+^ dyshomeostasis has been validated also in cerebellar granule neurons isolated from *mdx* mice [20]. Moreover, the presence of a chronic overload of Ca^2+^ with consequent cytotoxic effect on glutamatergic neurons could be correlated to degenerative neurological diseases. The current study is the first to show that Ca^2+^ dyshomeostasis occurs also in DMD sensorial neurons, as occurs in altered muscle. The elevation in [Ca^2+^]i appears to be due to an increase of Ca^2+^ release from intracellular stores such as sarco/endoplasmic reticulum (SR/ER) [21]. The same cellular dysfunctions were associated with a spatial learning deficit in *mdx* mice [22]. It is well established that disruption of intracellular Ca^2+^ homeostasis in neurons causes learning and memory dysfunctions, metabolic derangements, and cell death as it has been shown in several chronic diseases [22]. Loss and/or reduction of Dp71 and DAPs are thought to contribute to severity of mental retard [23]. Interestingly, we found that Dp71 co-localized with SERCA2, the main brain protein responsible for the removal of intracellular Ca^2+^, suggesting that functional alteration identified in DMD neurons could be related to Dp71 deficiency in DMD patient with mental retard. Furthermore, the overexpression of SERCA2 in DMD neurons suggests a neuronal mechanism to restore Ca^2+^ level in cytosol. SERCA pump is dysfunctional in severely affected muscles of *mdx* mice [16], but nothing is known about its functionality in the brain.

In conclusion, the Dp71 partial ablation and disarrangement correlate with abnormal Ca2+ homeostasis in human neurons. This altered molecular pathway may potentially contribute to altered brain function and cognitive deficits in DMD patients. Further studies are needed to clarify whether SERCA2 overexpression or its functional activity are involved in the impairment of ER-Ca2+ handling that we reported in DMD neurons. Identification of this ion channel and improved understanding of its regulation appear essential to better understand the disease for a possible new therapeutic approach.

## Additional file

Additional file 1: Supplemental information. (DOCX 1681 kb)

## Abbreviations

AQP4: Water channel aquaporin-4
CPA: Cyclopiazonic acid
CTRL: Control
DAPs: Dp71-associated proteins
Dys: Dp427 dystrophin
ER: Endoplasmic reticulum
hiPSCs: Human-induced pluripotent stem cells
NFH: Neurofilament
PBMC: Peripheral blood mononuclear cells
SERCA2: Ca/ATPasic pump 2 -isoform of brain
TuJ-1: Neuron-specific class III β-tubulin
βDG: Beta-dystroglycan

## References

[1] Ervasti JM, Campbell KP (1991). Membrane organization of the dystrophin-glycoprotein complex. Cell.

[2] Desguerre I, Christov C, Mayer M (2009). Clinical heterogeneity of duchenne muscular dystrophy (DMD): definition of sub-phenotypes and predictive criteria by long-term follow-up. PLoS One.

[3] Jung D, Filliol D, Metz-Boutigue MH (1993). Characterization and subcellular localization of the dystrophin-protein 71 (Dp71) from brain. Neuromuscul Disord.

[4] Austin RC, Morris GE, Howard PL (2000). Expression and synthesis of alternatively spliced variants of Dp71 in adult human brain. Neuromuscul Dis.

[5] Bresolin N, Castelli E, Comi GP (1994). Cognitive impairment in Duchenne muscular dystrophy. Neuromuscul Dis.

[6] Tadayoni R, Rendon A, Soria-Jasso LE (2012). Dystrophin Dp71: the smallest but multifunctional product of the Duchenne muscular dystrophy gene. Mol Neurobiol.

[7] Allen DG, Gervasio OL, Yeung EW (2010). Calcium and the damage pathways in muscular dystrophy. Can J Physiol Pharmacol.

[8] van Westering TL, Betts CA, Wood MJ (2015). Current understanding of molecular pathology and treatment of cardiomyopathy in duchenne muscular dystrophy. Molecules.

[9] D'Aiuto L, Zhi Y, Kumar Das D (2014). Large-scale generation of human iPSC-derived neural stem cells/early neural progenitor cells and their neuronal differentiation. Organogenesis.

[10] D'Aiuto L, Prasad KM, Upton CH (2015). Persistent infection by HSV-1 is associated with changes in functional architecture of iPSC-derived neurons and brain activation patterns underlying working memory performance. Schizophr Bull.

[11] Carmosino M, Gerbino A, Schena G (2016). The expression of Lamin A mutant R321X leads to endoplasmic reticulum stress with aberrant Ca(2+) handling. J Cell Mol Med.

[12] Gerbino A, Bottillo I, Milano S (2017). Functional characterization of a novel truncating mutation in Lamin A/C gene in a family with a severe cardiomyopathy with conduction defects. Cell Physiol Biochem.

[13] Robin G, Berthier C, Allard B (2012). Sarcoplasmic reticulum Ca2+ permeation explored from the lumen side in *mdx* muscle fibers under voltage control. J Gen Physiol.

[14] Annese T, Corsi P, Ruggieri S (2016). Isolation and characterization of neural stem cells from dystrophic *mdx* mouse. Exp Cell Res.

[15] Li Y, Zhang C, Xiong F (2008). Comparative study of mesenchymal stem cells from C57BL/10 and *mdx* mice. BMC Cell Biol.

[16] Gehrig SM, van der Poel C, Sayer TA (2012). Hsp72 preserves muscle function and slows progression of severe muscular dystrophy. Nature.

[17] Turner PR, Westwood T, Regen CM (1988). Increased protein degradation results from elevated free calcium levels found in muscle from *mdx* mice. Nature.

[18] Altamirano F, Perez CF, Liu M (2014). Whole body periodic acceleration is an effective therapy to ameliorate muscular dystrophy in *mdx* mice. PLoS One.

[19] Mijares A, Altamirano F, Kolster J (2014). Age-dependent changes in diastolic Ca(2+) and Na(+) concentrations in dystrophic cardiomyopathy: role of Ca(2+) entry and IP3. Biochem Biophys Res Commun.

[20] Hopf FW, Steinhardt RA (1992). Regulation of intracellular free calcium in normal and dystrophic mouse cerebellar neurons. Brain Res.

[21] Bowman CL, Gottlieb PA, Suchyna TM (2007). Mechanosensitive ion channels and the peptide inhibitor GsMTx-4: history, properties, mechanisms and pharmacology. Toxicon.

[22] Lopez JR, Kolster J, Uryash A, Estève E, Altamirano F, Adams JA. Dysregulation of Intracellular Ca2+ in Dystrophic Cortical and Hippocampal Neurons. Mol Neurobiol. 2018;55(1):603-18. 10.1007/s12035-016-0311-7 27975174

[23] Daoud F, Candelario-Martinez A, Billard JM (2008). Role of mental retardation-associated dystrophin-gene product Dp71 in excitatory synapse organization, synaptic plasticity and behavioral functions. PLoS One.

