# Supplementary material for: DP71 and SERCA2 alteration in human neurons of a Duchenne muscular dystrophy patient
Source: Stem Cell Res Ther. 2019 Jan 15;10:29. doi: 10.1186/s13287-018-1125-5 (PMC6334379; doi:10.1186/s13287-018-1125-5)
Supplement: Supplementary file 1 — Supplemental information. (DOCX 1681 kb) [file 13287_2018_1125_MOESM1_ESM.docx]

**Additional file 1**

**SUPPLEMENTAL EXPERIMENTAL PROCEDURES**

**Case report**. 22-year-old patient born at the end of a normal pregnancy. No family history of neuromuscular disorders and no perinatal complications were reported. The patient soon exhibited motor developmental delay: he began to hold his head at 8 months; he acquired sitting position at 16 months and he started to walk at 3 years and 6 months. A clinical and genetic diagnosis of Duchenne dystrophy was made (Figure S1). Physical and neurological examination showed proximal muscle symmetrical weakness, Gower positive sign, waddling gait, calf hypertrophy, deep tendon hyporeflexia and initial signs of lordosis. Serum muscular enzymes levels were significantly high (CPK 4000U/I, AST 55 U/I, ALT 78U/l, LDH 300 U/I) and genetic analyses for DMD were performed, confirming the clinical diagnosis.

The neuro-linguistic development was also delayed. At the age of 14 a diagnosis of Intellectual Disability (mild level) was made. At present, the patient shows a severe motor impairment, characterized by the loss of most motor abilities; he became wheelchair dependent by 10 years of age and later he developed a progressive respiratory failure that needed an assisted ventilation (initially nocturnally, with the subsequent addition of daytime ventilation). His total intelligence quotient (IQ), assessed with Wechsler Intelligence Scale for Children–Fourth Edition (WISC-IV), is 68, confirming the mild intellectual disability previously diagnosed. The Study was approved by the local Ethics Committee at the University of Bari Medical School, and the patient gave its informed consent in accordance with the declaration of Helsinki.

**Generation of DMD and control hiPSCs.** We have collected samples of PBMCs from a DMD patient and a healthy donor. PBMCs were reprogrammed to hiPSCs by nucleofection with three episomal plasmids encoding human L-MYC and LIN28, SOX2 and KLF4, and OCT4 combined with a short hairpin RNA for P53 (shP53)(1). iPS cell colonies displayed a typical ES cell-like colony morphology and stained positive for OCT4 and TRA-1-60. The expression of pluripotency genes *POU5F1* (Oct-4), *MYCL* (L-Myc); *KLF4*, *SOX2* and *LIN28* was higher in hiPSCs compared to patient fibroblasts while exogenous gene expression (from episomes) was silenced during amplification. Pluripotency was supported by the capability to differentiate into three germ layers in vitro, as confirmed by qRT-PCR showing expression  Sox17, FoxA2 and Gata4 (endoderm), Brachyury (T) and Eomes (mesoderm) and  β-III-tubulin, Sox1 and nesting (ectoderm). Differentiation capacity into three germ layers was also demonstrated by in vivo teratoma formation. iPSC had a numerically and structurally normal karyotype (46, XX).

**Neuronal Differentiation.** Human hiPSCs were maintained in supplemented mTeSR medium (Stem Cell Technologies) under standard conditions (5% CO_2_, 37°C, and 100% humidity). hiPSCs were differentiated to glutamatergic sensory neurons using a previously described protocol [[3](#_ENREF_3),[4](#_ENREF_4)]. Briefly, hiPSCs were cultured in neuronal precursor selection medium, followed by neuronal precursor expansion medium containing fibroblast growth factor 2 for generation of neural stem cells. After 5-7 days in culture, neural rosettes were identified, manually dissected and plated into low-attachment plates where embryoid body-like structures - denoted as neurospheres - emerged. On plating neurospheres into matrigel-coated plates, neural progenitor cells (NPCs) spread off from neurospheres. NPCs were then cultured in neurobasal medium supplemented with 2% B27 (Gibco), 10 ng/mL BDNF (PeproTech), 50 U/mL penicillin G, and 50 mg/mL streptomycin. Attached cells were differentiated in Neurobasal medium for 8-10 weeks. The culture medium was changed every day.

**Electron microscopy.** The hiPSCs and neurons cells were fixed in 0.1 M PBS 3% glutaraldehyde, were post-fixed with 1% osmium tetroxide, dehydrated in an ascending ethanol series and embedded in Epon 812. Semithin sections were cut and stained with toluidine blue. Thereafter, 80 nm ultrathin sections were cut with a diamond knife on an LKB V ultratome, stained with uranyl acetate and lead citrate and examined with a Zeiss EM109 electron microscope (Zeiss, Oberkochen, Germany).

**Dual immunofluorescence confocal laser scanning microscopy.** hiPSCs and neuronal cells were fixed in 4% PFA, rinse with PBS and then exposed to primary antibodies diluted in PBS with 0.2% BSA (Table S1). After, the cells were incubated for 45 min with the corresponding secondary antibodies at 37°C (Table S1), and following washing they were incubated for 20 min with 0.01% TO-PRO-3 (Invitrogen, Carlsbad, CA, USA) for nuclear staining, and mounted in Vectashield (Vector Laboratories, Burlingame, CA,USA).The cells were examined under a Leica TCS SP2 (Leica, Wetzlar, Germany) confocal laser scanning microscope using 40x and 63x objective lenses with either 1x or 2x zoom factors. A sequential scan procedure was applied during image acquisition of the two fluorophores. Confocal images were taken at 200 nm intervals through the z axis of the section. Images from individual optical planes and multiple serial optical sections were analyzed, digitally recorded, and stored as TIFF files using Adobe Photoshop software (Adobe Systems Inc. San Jose, CA, USA).

**Morphometric analysis**. Morphometric analysis was performed by two independent observers on ten randomly selected fields observed at 63x magnification for immunofluorescence reactions and at 7000x magnification for ultramicroscopic sections by using Cell^F as image analysis software (Olympus Italia, Rozzano, Italy).

**Real Time PCR.** RNA was extracted from cells using an RNeasy Mini Kit (Qiagen) and then used to synthesize the first-strand c-DNA with the IScriptcDNA Synthesis kit (Bio-Rad Laboratories, Hercules, CA, USA), according to the manufacturer’s instructions. cDNA was amplified with the iTaq SYBR Green Supermix (Bio-Rad Laboratories, Hercules, CA, USA). PCR amplification and Real-time detection was performed with the Chromo4 Real-Time PCR Detection System (Bio-Rad Laboratories). The expression of mRNA for Dp71, AQP4, Dp427, β-DG and SERCA was evaluated by Real-Time PCR and samples were normalized to *cyclophylin A* as housekeeping gene. The primer sequences are reported in Table S2 . The analysis was performed with Bio-Rad CFX manager 3.1 software.

**Western blotting**. Cells from control and DMD iPSCs ad Neurons were homogenized in a lysis buffer (20 mM Tris-HCl pH 7.5, 1% (v/v) Triton X-100, 1% (v/v) NP-40, 2 mM MgCl2, 5 mM EDTA, 150 mM NaCl, 0.2 mM phenylmethylsulfonyl fluoride, 1 mM NaVO4, 10 mM NaF and protease inhibitors) and incubated for 3 time at 20 °C 10 min. After centrifugation at 14,000 rpm for 20 min, the protein concentration of the supernatant was determined using the detergent-compatible Bio-Rad DC protein assay (Bio-Rad Laboratories). For immunoblotting, 30 µg per lane of protein extract was solubilized in Laemmli buffer, boiled at 90 °C for 5 min and resolved on a 7.5-12% polyacrylamide gel; thereafter, the proteins were electrotransferred to a nitrocellulose membrane (Amersham Bioscience, Buckinghamshire, UK). Blots were blocked with PBS blocking buffer containing 5% nonfat dry milk for 1 h and incubated overnight at 4 °C with the following primary antibodies: Dys, SERCA2, Dp71, βDG, AQP4 and βActin (Table S1). After the primary antibody treatment, the membranes were washed 4x5 min each at room temperature in PBS with 0.1% Tween-20 before the addition of secondary antibodies. PBS and 0.1% Tween-20-diluted secondary antibodies (anti-mouse and rabbit) were IRDye labelled (800CW) (LI-COR Biosciences, Lincoln, NE, USA). For immunoblotting analysis, the LI-COR Odyssey infrared imaging system was used (LI-COR). The western blot images were analyzed by imaging densitometry using ImageJ compared with actin and expressed as optical density mm2 . The images are representative of three independent experiments performed in triplicate.

**Electrophysiological measurements.** Both Control and Patient’s iPSC-derived glutamatergic neurons, plated on 35 mm culture dishes, were recorded at room temperature. Whole cell experiments were performed with a Multiclamp 700B amplifier (Axon CNS-Molecular Devices, Sunnyvale, CA, USA) connected to an Axon Digidata 1500 (Axon Instrument-Molecular Devices, Sunnyvale, CA, USA). Currents were sampled at 10kHz and low-pass filtered at 5 kHz.

The bath external solution contained in mM: 140 NaCl, 2.8 KCl, 1 CaCl2, 0.01 EDTA, 10 Hepes. pH was corrected to 7.2, osmolarity 280±5 mmol/Kg.

Patch pipettes, pulled with the P-1000 Pipette puller (SUTTER INSTRUMENT, Novato, CA, USA), had a tip resistance of 5-9 MΩ when filled with the internal pipette solution. The internal pipette solution contained in mM: 130 K-gluconate, 10 NaCl, 1 CaCl2, 1 EGTA, 10 Hepes, 2 ATP-Na2, 2 MgCl2. pH was adjusted to 7.2, osmolarity 280±5mmol/Kg.

Data were recorded using AxoScope 10.4 (Molecular Devices, Sunnyvale, CA, USA) and analyzed using pClamp 10.4 (Molecular Devices, Sunnyvale, CA, USA).

The currents were recorded from a holding potential of -85 mV with depolarizing steps of 400 ms from -‍100mV to 20mV. Action potentials were evoked using 500ms depolarizing steps of current from -‍100 pA to 80 pA with a holding current of -60pA.

**Intracellular Ca^2+^ measurements.** Cells were seeded on matrigel-coated glass coverslips (Ø 35 mm). Ringer’s Solution was used to perfuse cells during the experiment containing 140 mM NaCl, 2.8 KCl, 1 mM MgCl2, 10 mM Hepes, 5 mM Glucose, 1.0 mM CaCl2, pH 7.4. Neurons were stimulated with a variety of drugs as described in the results, including cyclopiazonic acid (CPA) and ionomycin (both from Sigma-Aldrich, St. Louis, USA). Cells were loaded with 2-4 µM Fluo-4 (Thermo Fisher Scientific, Waltham, MA, USA) 25 min at 37°C in DMEM. Coverslips with dye-loaded cells were mounted in a perfusion chamber (FCS2 Closed Chamber System, BIOPTECHS, Butler, U.S.A.) and measurements were performed using an inverted microscope (Nikon Eclipse TE2000-S microscope) equipped for single cell fluorescence measurements and imaging analysis. The sample was illuminated through a 40X oil immersion objective (NA = 1.30). The Fluo-4 loaded sample excited at 488/20 every 5 seconds. Emitted fluorescence was passed through a dichroic mirror, filtered at 520 nm (Omega Optical, Brattleboro, VT, USA) and captured by a cooled CCD camera (CoolSNAP HQ, Photometrics, Tucson, AZ, USA). Fluorescence measurements were performed in regions corresponding to neuron cell body (soma) using Metafluor software (Molecular Devices, MDS Analytical Technologies, Toronto, Canada).

**SUPPLEMENTAL FIGURES**

**FIGURE S1**


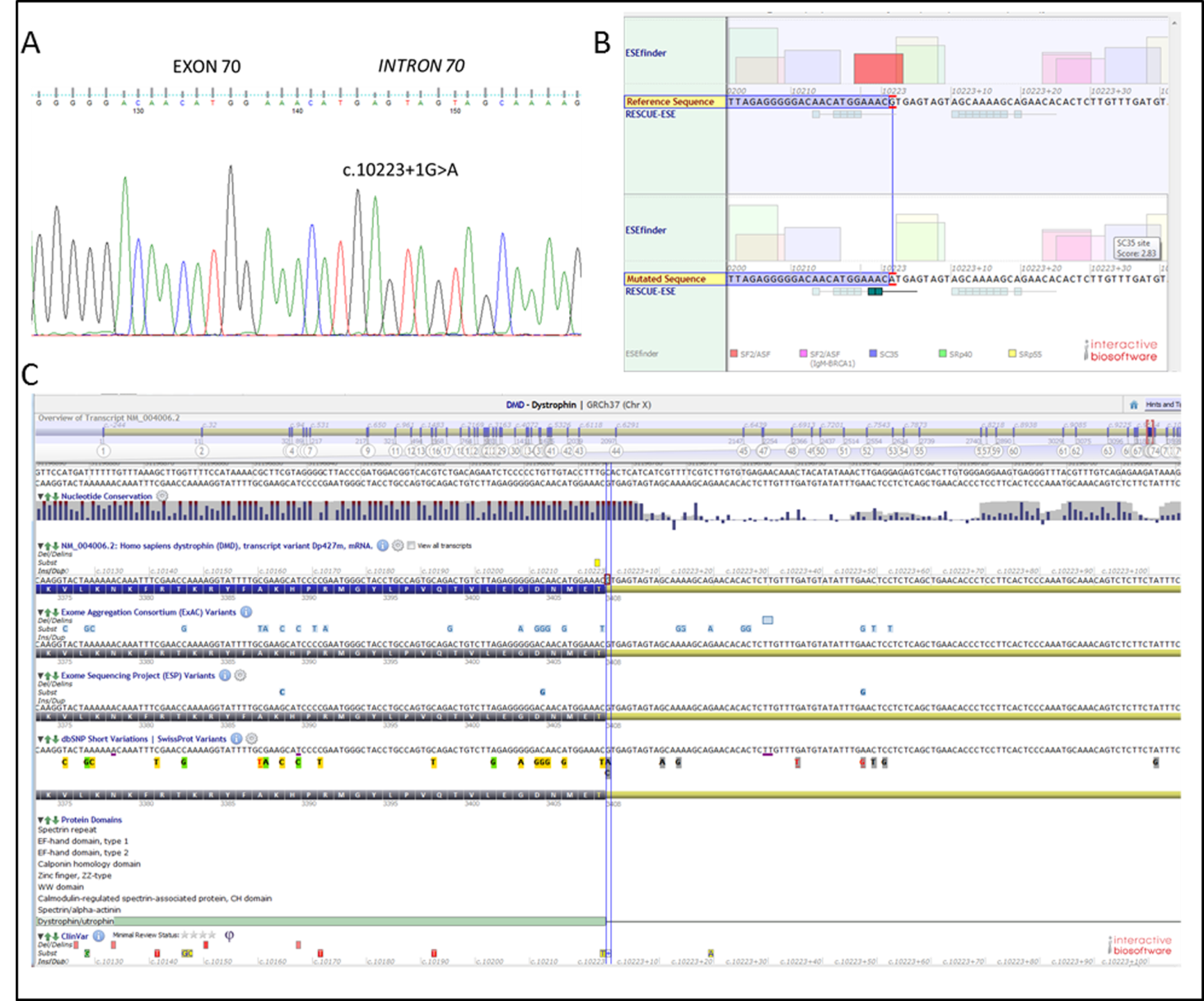


**FIGURE S2**


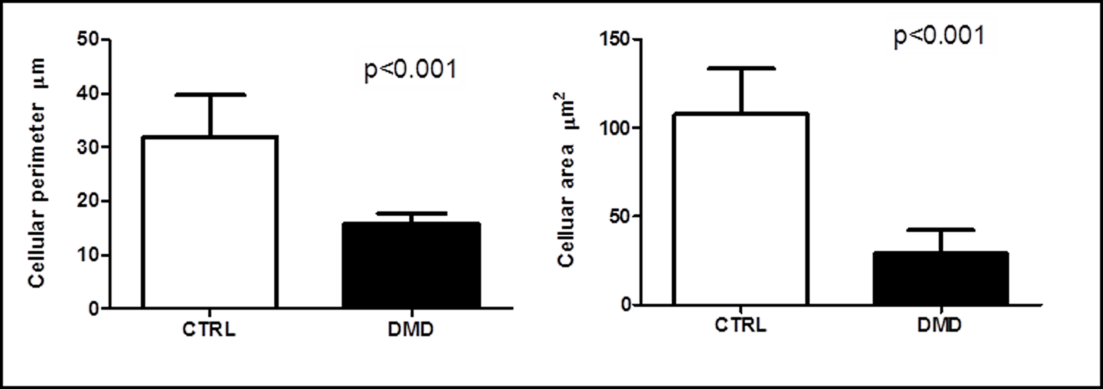


**FIGURE S3**


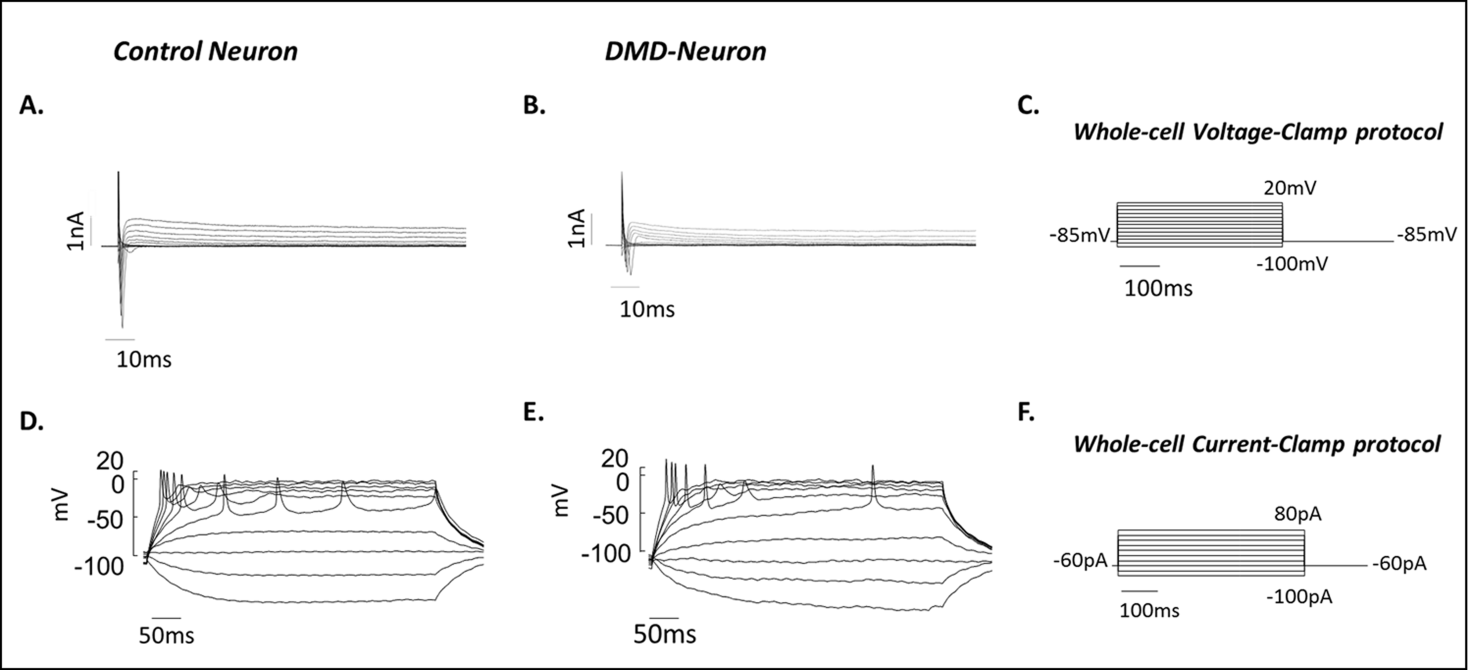


**FIGURE S4**

**
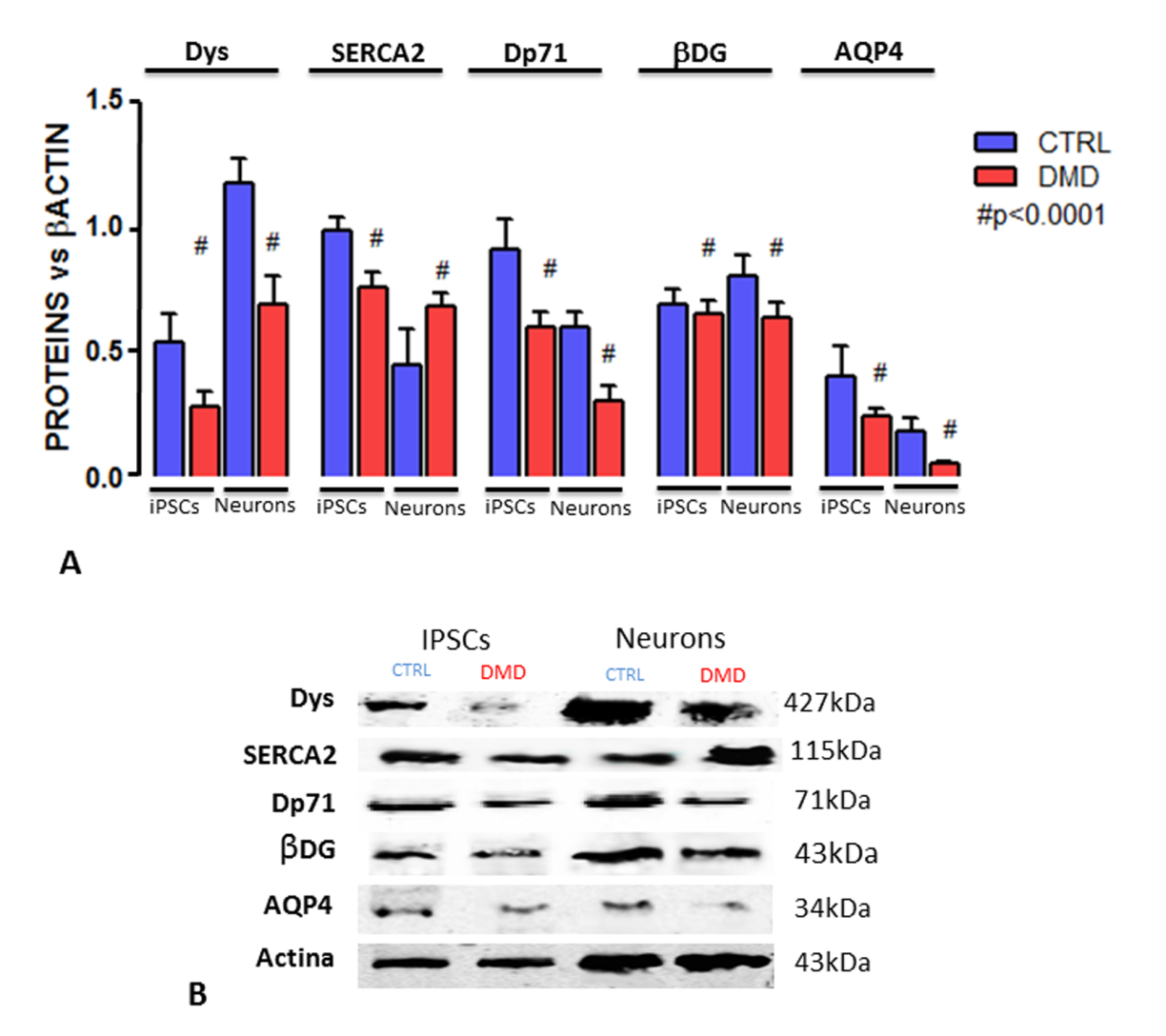
**

**SUPPLEMENTAL FIGURE TITLE AND LEGENDS**

**Figure S1.** **Genetic analysis.** **(A)** Electropherogram showing the intron variant in the splicing donor site c.10223 + 1G> A(being an X linked gene and male XY probing is only one allele). **(B)** Prediction of the binding of transcription factors: variant c.10223 + 1G> A abolishes the binding site for the SF2 / ASF factor. **(C)** Displaying the DMD gene with the Alamut software: the base (G) is highlighted between two blue lines and in the patient it is mutated (G> A).

**Figure S2.** **Morphometric analysis**. Morphometric analysis shows a significative reduction of iPSCs perimeter and surface area in DMD compared with control one.

**Figure S3.** **Current recorded in neurons differentiated by hiPSCs.** Currents recorded in the differentiated control neurons **(A)** and in the DMD-neurons **(B)** consisting of a sodium inward component and of a potassium outward component. The currents are recorded in Whole-cell Voltage Clamp, depolarizing the cells from -100mV to 20mv, from an holding potential of -85mV **(C)**. Evoked action potentials are recorded in differentiated control neurons **(D)** and in differentiated patient’s neurons **(E)**, using a current clamp protocol **(F)** consisting of depolarizing steps of current from -100pA to 80pA, from an holding current of -60 pA.

**Figure S4. Western blotting analysis. (A)** Immunoblotting of Dys, SERCA2 , Dp71, β-DG, and AQP4 in iPSCs and Neurons homogenates of ctrl and DMD patient. **(B)** Quantification of the bands reveals a significantly lower level of Dys, Dp71, β-DG, and AQP4 proteins in DMD iPSCS and Neurons compared to ctrl, while SERCA2 protein decreases in DMD iPSCS but increases in DMD Neurons.

**SUPPLEMENTAL TABLES**

**Table S1. Antibodies used for immunofluorescence.**

| Protein | Catalog number | Species | IF  Dilution | WB Diluition | Source |
| --- | --- | --- | --- | --- | --- |
| AQP4 | sc-20812 | rabbit | 1:25 | 1:100 | Santa Cruz Bio. |
| βDG | NCL-b-DG | mouse | 1:500 | 1:50 | Novocastra |
| Dp71 | MANDRA1 | mouse | 1:15 | 1:100 | Sigma-Aldrich |
| Dys | ab131315 | rabbit | 1:50 | 1:100 | Abcam |
| SERCA2 | Ab150435 | rabbit | 1:100 | 1:100 | Abcam |
| TuJ-1 | MAB1195 | mouse | 1:50 |  | R&D Systems |
| NFH | Ab8135 | rabbit | 1:500 |  | Abcam |
| VGLUT-1 | 135303 C5 | mouse | 1:500 |  | Synaptic-Systems |
| Alexa fluor 488 antimouse | A11017 | goat | 1:300 |  | Invitrogen |
| Alexa fluor 555 antirabbit | A21430 | goat | 1:900 |  | Invitrogen |
| Alexa fluor 488 antirabbit | A11034 | goat | 1:300 |  | Invitrogen |
| Alexa fluor 555 antimouse | A32727 | goat | 1:900 |  | Invitrogen |
| IRDye 800CW antirabbit | 926-32211 | Goat |  | 1:7000 | LI-COR |
| IRDye 800CW antimouse | 926-32210 | Goat |  | 1:7000 | LI-COR |

AQP4 = water channel aquaporin-4; βDG = beta-dystroglican; Dys = Dp427 dystrophin; SERCA2 = Ca/ATPasic pump  2; TuJ-1 = Neuron-specific class III β-tubulin ; NFH = neurofilament VGLUT-1 = vesicular glutamate transporter 1

**Table S2. Biorad primer PCR**

| Gene | code |
| --- | --- |
| AQP4 | qHsaCID0009581 |
| DG | qHsaCED0046978 |
| Dp71 | 10025220 |
| Dys | qHsaCID0010707 |
| SERCA2 | qHsaCID0011088 |
| Ciclophylin A | qHsaCED0038620 |

AQP4 = water channel aquaporin-4; βDG = beta-dystroglican; Dys = Dp427 dystrophin; SERCA2 = Ca/ATPasic pump

**REFERENCES**

1. Okita K, Matsumura Y, Sato Y*, et al.* A more efficient method to generate integration-free human iPS cells. *Nature methods* 2011; **8**: 409-412.

2. D'Aiuto L, Prasad KM, Upton CH*, et al.* Persistent infection by HSV-1 is associated with changes in functional architecture of iPSC-derived neurons and brain activation patterns underlying working memory performance. *Schizophrenia bulletin* 2015; **41**: 123-132.

3. D'Aiuto L, Zhi Y, Kumar Das D*, et al.* Large-scale generation of human iPSC-derived neural stem cells/early neural progenitor cells and their neuronal differentiation. *Organogenesis* 2014; **10**: 365-377.
